# *unc-42* regulates the expression of ASH terminal fate markers

**DOI:** 10.17912/micropub.biology.000114

**Published:** 2019-05-15

**Authors:** Jordan F. Wood, Denise M. Ferkey

**Affiliations:** 1 Department of Biological Sciences, University at Buffalo, The State University of New York, Buffalo, NY USA 14260

**Figure 1. unc-42 regulates terminal gene battery expression in the ASH nociceptors:**
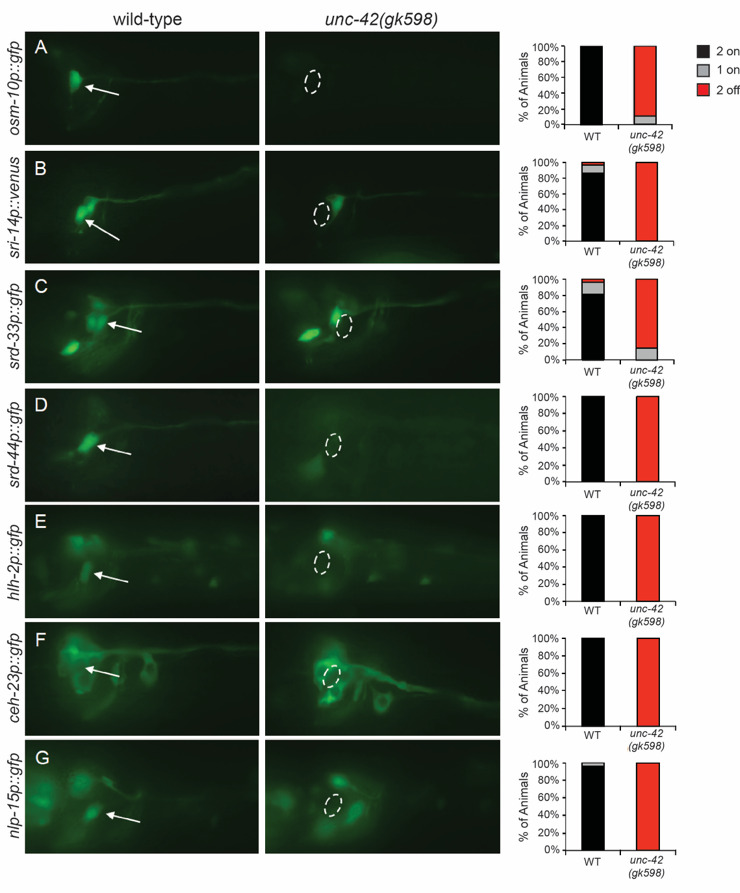
*unc-42* function is required for the expression of ASH terminal differentiation gene marker expression specifically in the ASH neuron pair; expression in other neurons did not appear to be affected. The ASH neurons were identified by positional labeling with the lipophilic dye DiD, as previously described (Perkins *et al.* 1986). (A-G) Images of GFP reporter expression were obtained with a Zeiss Axio Imager Z1 Microscope, high resolution AxioCam MRm digital camera and Zeiss AxioVision software. Left panels indicate ASH neurons in wild-type animals, indicated by arrows. Right panels show the lack of expression in the ASH neurons of *unc-42(gk598)* mutant animals, indicated by dashed circles. Bar graphs show the percentage of animals examined showing GFP marker expression in 2, 1 or neither of the ASH neurons. A mixed age population of n > 40 animals was examined for each transgene.

## Description

The genes encoding transcription factors that initiate the terminal differentiation programs within individual neurons have been termed “terminal selectors” (Hobert 2008). They are considered master regulators of neuronal identity because they integrate upstream lineage inputs to ultimately drive (directly and/or indirectly) the expression of all terminal differentiation features of a neuron (Hobert 2008; Bertrand and Hobert 2010). The paired-like homeodomain transcription factor UNC-42 has been proposed to function as the terminal selector in the developmental specification of the ASH polymodal nociceptors in *C. elegans* (Baran *et al.* 1999). Consistent with the role of a terminal selector, *unc-42(e419)* loss-of-function mutants were shown to lack the expression of the putative chemoreceptor genes *sra-6* and *srb-6* specifically in the ASHs, while, for example, *srb-6* expression in the ADL and ADF sensory neurons was unaffected (Baran *et al.* 1999). *unc-42(e419)* mutants also lack ASH expression of several other terminal identity markers: three Gα encoding genes (*gpa-11, gpa-13* and *gpa-15*), the neuropeptide encoded by *flp-21*, and the *eat-4* vesicular glutamate transporter (Serrano-Saiz *et al.* 2013). Furthermore, loss of UNC-42 function disrupted behavioral responses to high osmolarity and nose touch (Baran *et al.* 1999), which are both detected primarily by the ASHs (Bargmann *et al.* 1990; Kaplan and Horvitz 1993). However, the severity of the nose touch defect was likely due to developmental defects in both the ASH sensory neurons as well as some downstream interneurons, where altered glutamate receptor expression accompanies loss of UNC-42 (Baran *et al.* 1999; Brockie *et al.* 2001).

**Table:** 

	Identity marker		Expression affected in *unc-42(gk598)* mutant background
ASH nociceptive neuron	High osmolarity detection	*osm-10*	YES
	GPCR	*sra-6**sri-14**srd-33**srd-44**srd-10**srz-1*	YES*YESYESYESYES (partial)NO
	Transcription factor	*hlh-2**ceh-23**nhr-79*	YESYESNO
	Neuropeptide	*nlp-15**nlp-3*	YESNO
	TRP channel	*osm-9**ocr-2*	NONO
	Axon guidance	*unc-40**sax-3*	NONO

**Table 1**: Summary of the effect of *unc-42(gk598)* on ASH terminal marker gene expression *sra-6 expression in ASH was previously reported to be affected by *unc-42(e419)* (Baran et al. 1999).

Using the *unc-42(gk598)* allele, we confirmed the role of UNC-42 in regulating ASH terminal markers, and identified seven additional genes whose ASH expression strongly depends upon UNC-42 (Figure 1 and Table 1). These genes encode OSM-10, additional predicted GPCRs, transcription factors and a neuropeptide. However, UNC-42 is unlikely to function as the sole terminal selector in ASH. For example, *srd-10* expression is only partially affected in the *unc-42(gk598)* mutants, with only ~20% of animals losing expression in one ASH (image not shown). In addition, ASH expression of some genes (*srz-1*, *nhr-79*, *nlp-3*, *osm-9*, *ocr-2*, *unc-40* and *sax-3*) was found to be unaffected in *unc-42(gk598)* animals (Table 1). We note that several of these are either expressed broadly throughout the nervous system, or function in several sensory neurons. As such, they may not necessarily be constituents of the terminally differentiated gene battery of the ASHs. Combined, UNC-42 broadly regulates the expression of ASH identity markers, although additional transcription factors are likely to also contribute.

## Reagents

DiD was purchased from Molecular Probes (Invitrogen).

The VC1444 *unc-42(gk598)* strain was generated by the *C. elegans* Reverse Genetics Core Facility at the University of British Columbia, which is part of the International *C. elegans* Gene Knockout Consortium. The *gk598* allele contains a 1430 basepair deletion (898 basepairs of 5’ UTR sequence, exon 1 and 481 basepairs of intron 1). VC1444 was outcrossed 6x to N2 to generate FG498 for use in this study. Some of the strains used in this study were obtained from the *Caenorhabditis* Genetics Center, which is funded in part by the National Institutes of Health – National Center for Research Resources.

Strains used in this study include: N2 Bristol wild-type, FG498 *unc-42(gk598)*, CX3465 *kyIs39 [sra-6::gfp + lin-15(+)]*, HA1695 *rtIs27 [osm-10p::gfp]*, PY5163 *[oyEx srd-44p::gfp, coelRFP] line 1*, NK885 *unc-119(ed4);qyIs174 [hlh-2p::gfp::hlh-2 + unc-119(+)]*, CX2565 *kyIs4 lin-15A(n765) [ceh-23p::unc-76::gfp + lin-15(+)]*, RW10748 *unc-119(ed3); zuIs178; stIs10024; stIs10549 [stIs10549 = nhr-79::H1-wCherry + unc-119(+)]*, HA357 *rtEx251 [nlp-15p::gfp + lin-15(+)]; lin-15B lin-15A(n765)*, AL132 *icIs132 [unc-40::gfp]*, HA341 *lin-15; rtEx235 [nlp-3::gfp]*, LX990 *lin-15B lin-15A(n765); vsEx494 [ocr-2p::gfp::ocr-2 3’utr + lin-15(+)]*, CX3716*lin-15B lin-15A(n765) kyIs141 [osm-9::gfp5 + lin-15(+)]*, IC692 *quEx162 [sax-3p::gfp + (pRF4) rol-6]*. Transgenes were examined in the wild-type and*unc-42(gk598)* background. For the generation of extrachromosomal arrays, germline transformations were performed as previously described (Mello *et al.* 1991). Plasmids injected for analysis include: *sri-14p::venus*, pFG258 *srd-33p::gfp* and pFG233 *srd-10p::gfp*. Transgenic lines were examined in the N2 wild-type background, and at least one array was chosen to be crossed into the *unc-42(gk598)* background for comparison. Strains generated in our lab for this study have not been sent to the CGC.
